# Could TnI level on admission predict function and infarct size in STEMI patient treated with pPCI – CMR study

**DOI:** 10.1186/1532-429X-11-S1-P258

**Published:** 2009-01-28

**Authors:** Dorota Piotrowska-Kownacka, Lukasz Kownacki, Grzegorz Opolski, Leszek Krolicki, Olgierd Rowinski

**Affiliations:** Medical University of Warsaw, Warsaw, Poland

**Keywords:** Infarct Size, Scar Size, Cardiac Coil, Segment Elevation Myocardial Infarction, Ventricle Function

## Introduction

Pain to balloon time could have significant error due to often imprecise pain onset time. We hypothesize that TnI level measured on admission could be easy monitored, independent predictor of infarct size and left ventricle function

## Purpose

The purpose of the study was to evaluate which of the two factors better correlates with LV function and delayed enhancement volume after successfully treated first ST segment elevation myocardial infarction (STEMI).

## Methods

34 patients with first anterior STEMI who underwent successful primary PCI (TIMI III) were included into the study. Cardiovascular MRI was performed on 1.5 T scanner at discharge, using 32-channel cardiac coil. Viability was evaluated on contrast-enhanced images acquired with the use of an inversion-recovery segmented gradient-echo sequence 20 minutes after contrast injection. Infarct size and LV function were analyzed quantitatively on AW workstation using MASS software.

## Results

Mean pain to balloon time was 3.8 ± 2.6 h (ranged between 1 do 14 h). Median TnI level on admission was 0.6 ng/ml (ranged between 0.0 and 25.55 ng/ml). Mean LVEF was 40.7% ± 10.9, scar size was 48 ml ± 23 or 31% ± 13.7 as a percent of LV volume. Linear regression analyses revealed mild correlation between pain to balloon time and LV EF (R = 0.555; p < 0.001) and low but statistically significant with infarct size as a % of LV volume (R = 0.34; p = 0.048). No correlation was found between TnI level on admission and infarct size or LVEF.

## Conclusion

Patients with longer pain to balloon time have worse LV EF at discharge.

TnI level on admission could not be a predictor of infarct size or early ejection fraction.

